# Supplementary material for: Chromothripsis during telomere crisis is independent of NHEJ, and consistent with a replicative origin
Source: Genome Res. 2019 May;29(5):737–49. doi: 10.1101/gr.240705.118 (PMC6499312; doi:10.1101/gr.240705.118)
Supplement: Supplemental Material [file supp_gr.240705.118_Supplemental_file_1.zip › contigs/annotated_contigs/DB53/contig.3.DB53_length_639_mean_cov_25.1408450704.docx]

**DB53_length_639_mean_cov_25.1408450704**

```
AACAAAATATGTATACAACTTATCTGATAAAAGCCACAAAATTCTGATGAAAGAAATCAGAGATGATCTAAATAAATGGAGAAATATTC
 >chr8:85422028-85422453 - E=5e-238 p=1e-02
CATGTTCATAGATATTGCCAAGATACCAACTTTTCCCAACTTTATCCATAGATTCAATGCAATCTCAATAAAAATTCCAGGAAATTACT

TTGTGGATATCAACAAAATGATTCCTAACTTTACATGGAGAGGCTAAGTTCCCAGAATGGGCAACCTAATATTGAAGAACAAAGTTGGA

AGGGACACAACCCAACTTTGAGACTCAATATAAAGCTACAGTAATAAAGACTGGTATTGGCAAAAGAATGGAGAAATAGATTAGTGGTA

CAGAATAGAGAGCTCAGAAATACACCCATATGAACACAGTCAACTGATCTTTGAAAAAGTAGCAAAAGC|C|ATGATTATTATAGCCTT
                                                                       >chr8:81611264-8
TCCAGTAAAAGATTTTATTTTCAGACATTATTCCTTATTTTAAGAATATCACAAGTTTTTAAAAGTGACTGAACTTGGTCTAGACAGCA
1611429 - E=1e-87
GATTTCAATAGTTGCTAAGAAACTAGATTAGTGCTAATCTAAGGAATATATTATTCATT|CGTAATGAAT|GACATGTTTAGATACACA
                                                                      >chr8:78555650-78
AATAGTTATCAT|GTTTTTAC555680 + E=3e-06
```
